# Effect of Gelatin-Based Hemostats on Fibroblasts and Relevant Growth Factors in Wound Healing

**DOI:** 10.3390/gels9060504

**Published:** 2023-06-20

**Authors:** Waseem Garabet, Polina Shabes, Katharina Henrika Wolters, Julian-Dario Rembe, Wiebke Ibing, Markus Udo Wagenhäuser, Florian Simon, Hubert Schelzig, Alexander Oberhuber

**Affiliations:** 1 Department of Vascular and Endovascular Surgery, University Hospital of Düsseldorf, 40225 Düsseldorf, Germany; polina.shabes@med.uni-duesseldorf.de (P.S.); katharinahenrika.wolters@med.uni-duesseldorf.de (K.H.W.); julian-dario.rembe@med.uni-duesseldorf.de (J.-D.R.); wiebke.ibing@med.uni-duesseldorf.de (W.I.); markus.wagenhaeuser@med.uni-duesseldorf.de (M.U.W.); florian.simon@med.uni-duesseldorf.de (F.S.); hubert.schelzig@med.uni-duesseldorf.de (H.S.); 2 Department of Vascular and Endovascular Surgery, University Hospital of Münster, 48149 Münster, Germany; alexander.oberhuber@ukmuenster.de

**Keywords:** hemostats, wound healing, fibroblast, exposure, gelatin, cytokine

## Abstract

Gelatin-based hemostats have been used in various surgical fields and showed advantageous effects on central aspects of wound healing when compared to cellulose-based hemostats. Nevertheless, the influence of gelatin-based hemostats on wound healing has not been fully explored yet. Hemostats were applied to fibroblast cell cultures for 5, 30, 60 min, 24 h, 7 and 14 days and measurements were taken at 3, 6, 12, 24 h and 7 or 14 days, respectively. Cell proliferation was quantified after different exposure times and a contraction assay was conducted to measure the extent of the extracellular matrix over time. We further assessed quantitative levels of vascular endothelial growth factor and basic fibroblast growth factor using enzyme-linked immunosorbent assay. Fibroblast counts decreased significantly at 7 and 14 days independent of the application duration (*p* < 0.001 for 5 min application). The gelatin-based hemostat did not have a negative impact on cell matrix contraction. After application of gelatin-based hemostat, the basic fibroblast growth factor did not change; yet, the vascular endothelial growth factor significantly increased after a prolonged 24 h application time when compared to controls or to a 6 h exposure (*p* < 0.05). Gelatin-based hemostats did not impair contraction of the extracellular matrix or growth factor production (vascular endothelial growth factor and basic fibroblast growth factor), while cell proliferation diminished at late time points. In conclusion, the gelatin-based material seems to be compatible with central aspects of wound healing. For further clinical assessment, future animal and human studies are necessary.

## 1. Introduction

Wound healing disorders after surgery are a common clinical problem. Fibroblasts play an essential role in controlling the wound-healing process in its different stages, such as the proliferation, contraction and production of regulatory growth factors [1,2,3].

During the proliferation phase of wound repair, the formation of granulation tissue occurs, which is mainly facilitated by fibroblast migration, angiogenesis, and wound contraction [2].

The synthesis and reorganization of essential components of the extracellular matrix (ECM) are primarily attributed to the role of fibroblasts [1,2].

During the second week of the wound-healing process, provided undisturbed regeneration progress, fibroblasts undergo differentiation into myofibroblasts, which secrete cytokines and simultaneously use proteases to remove damaged collagen and other cell detritus from the extracellular matrix (ECM) [1]. This complex process takes place throughout the proliferation and remodeling phase, leading to contraction of the collagen matrix and eventually resulting in wound closure [4,5,6,7,8]. Therefore, fibroblasts (and endothelial cells) majorly influence extracellular matrix remodeling and development of granulation tissue [2,9].

Various cytokines, growth factors, and their receptors play an important role in wound healing [1,2,10,11].

VEGF (vascular endothelial growth factor) as part of the PDGF (platelet-derived growth factor) family plays a crucial role in angiogenesis, which, in turn, is obligatory for a sustainable granulation tissue and is able to evoke proliferative and chemotactic reactions in endothelial cells [1,12,13]. FGF-b stimulates collagen synthesis, wound contraction, epithelialization, angiogenesis and fibronectin as well as proteoglycan synthesis [1,14,15].

Edg-1, the G protein-coupled receptor for sphingosine-1-phosphate, has been identified as a critical factor in vascular maturation [16]. Furthermore, the role of lysophospholipid mediators in regulating fibroblast functions and their potential involvement in wound healing has been highlighted in a study by Watterson et al. [17].

The use of hemostats is a standard procedure in surgery. Among commonly used cellulose-based hemostats [18,19], gelatin-based hemostats have been introduced in several animal and human (in vitro and in vivo) studies [20,21,22,23,24,25,26,27,28,29,30]. To set a clinical example, a thrombin–gelatin matrix has shown very potent hemostatic effects even in severe intraoperative bleeding situations, such as those presented in a prospective study in over 5000 spine surgery patients [31].

Gelatin has been historically used as an antipyretic and hemostatic medication and as a component in wound dressings [32]. Since their introduction in the 1940s, hemostats based on gelatin have undergone minimal changes and have been employed in various types of surgical procedures [23,33].

Gelatin works as a hemostatic agent by acting as a mechanical tamponade or a “hemostatic plug”, and it is generally absorbable [33,34,35]. Resorption times ranging from two to six weeks have been reported [24,34].

In recent studies, gelatin is widely used in wound dressings, either solely or in combination with different materials and/or chemical reagents (e.g., nitric oxide, silver, iron-containing nanozymes releasing hydroxyl radicals) resulting in positive effects on wound healing [36,37,38]. This is supported by in vitro studies, like an inflammatory model with gelatin/alginate-based microspheres that positively affected cytokines including bFGF and VEGF that play a central role in wound healing [39]. Furthermore, gelatin-based hydrogels are used as a skin substitute [40].

In our former in vitro study, the gelatin-based hemostat GELITA TUFT-IT (Gelita Medical, Eberbach, Germany) did not demonstrate a negative effect on wound-healing-related processes such as pH values, cytokine levels (TGF-β, TNF-α), fibroblast cell viability and migration when compared to cellulose-based hemostats, thus indicating a possible advantage of gelatin [19].

The aim of this study was to investigate the effect of the gelatin-based hemostat GELITA TUFT-IT (TUFT-IT) on fibroblasts in vitro. Fibroblast proliferation and contraction, as well as its influence on the expression of growth factors involved in wound healing, were investigated in the presence of TUFT-IT (VEGF, FGF-b). To mimic different application times during surgical procedures (ranging from minutes to being left in situ), the effects were investigated at various exposure times.

## 2. Results

### 2.1. Cell Proliferation

During the first 24 h, there was no significant difference between the groups with different application times of 5, 30, 60 min and 24 h, nor with permanent gelatin-based hemostat application when compared to controls. At later follow-up time points of 7 and 14 days, there was a significant cell number decrease independent of the application duration.

Regarding an application time of 5 min, the cell number decreased significantly after 7 and 14 days (*p* < 0.001). In terms of absolute numbers, the living cells decreased from initially 94.8 ± 14.0% to 14.8 ± 4.6% after 7 days and to 22.7 ± 11.8% after 14 days.

Additionally, permanent gelatin-based hemostat application led to a significant decrease in living cell numbers from initially 94.8 ± 14.0% to 30 ± 9.1% after 7 days and to 33.6 ± 3.3% after 14 days (*p* < 0.001).

**Figure 1:**
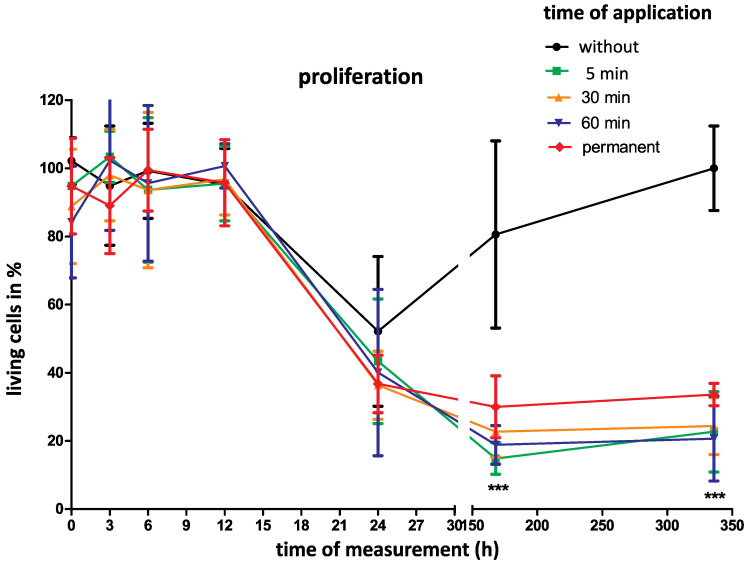
*** Significant proliferation decrease after 5, 30 and 60 min as well as after permanent gelatin-based hemostat application at 7 and 14 days (*p* < 0.001).

In the control group cell numbers only decreased to 80.5± 27.5% after 7 days. After 14 days, the cells recovered completely and increased to the initial cell numbers (Figure 1).

Fibroblast proliferation with and without gelatin-based hemostat application was assessed. Proliferation of the NHDF (normal human dermal fibroblast) cell line with and without gelatin-based hemostat application was measured at different time points (0, 3, 6, 12, 24, 7 d, 14 d) with different application times (5, 30, 60 min, permanent application). The control group comprised untreated cells. As a result, fibroblast proliferation remained unchanged in the control and application groups within the first 24 h. There was a significant difference between the application and control groups after 7 and 14 days with a proliferation decrease in the application group. There was no significant difference between the application groups themselves. (*** *p* < 0.001; two-way ANOVA with Bonferroni test).

### 2.2. Contraction Assay

**Figure 2:**
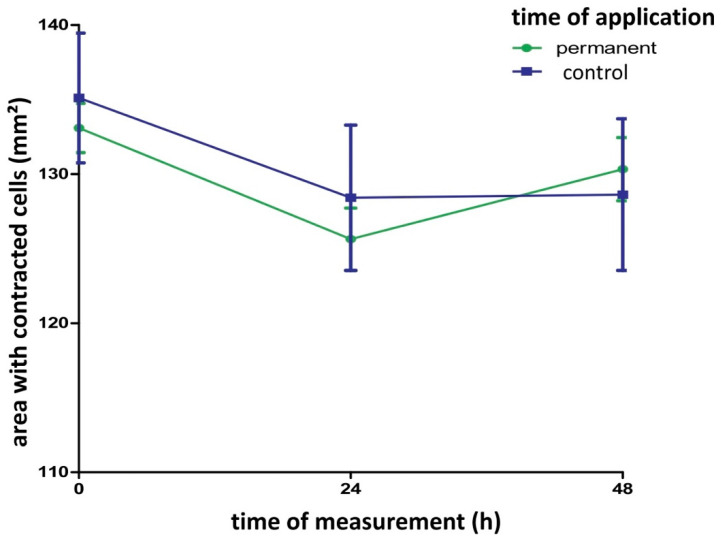
Contractile ability of the collagen matrix with and without gelatin-based hemostat application.

**Figure 3:**
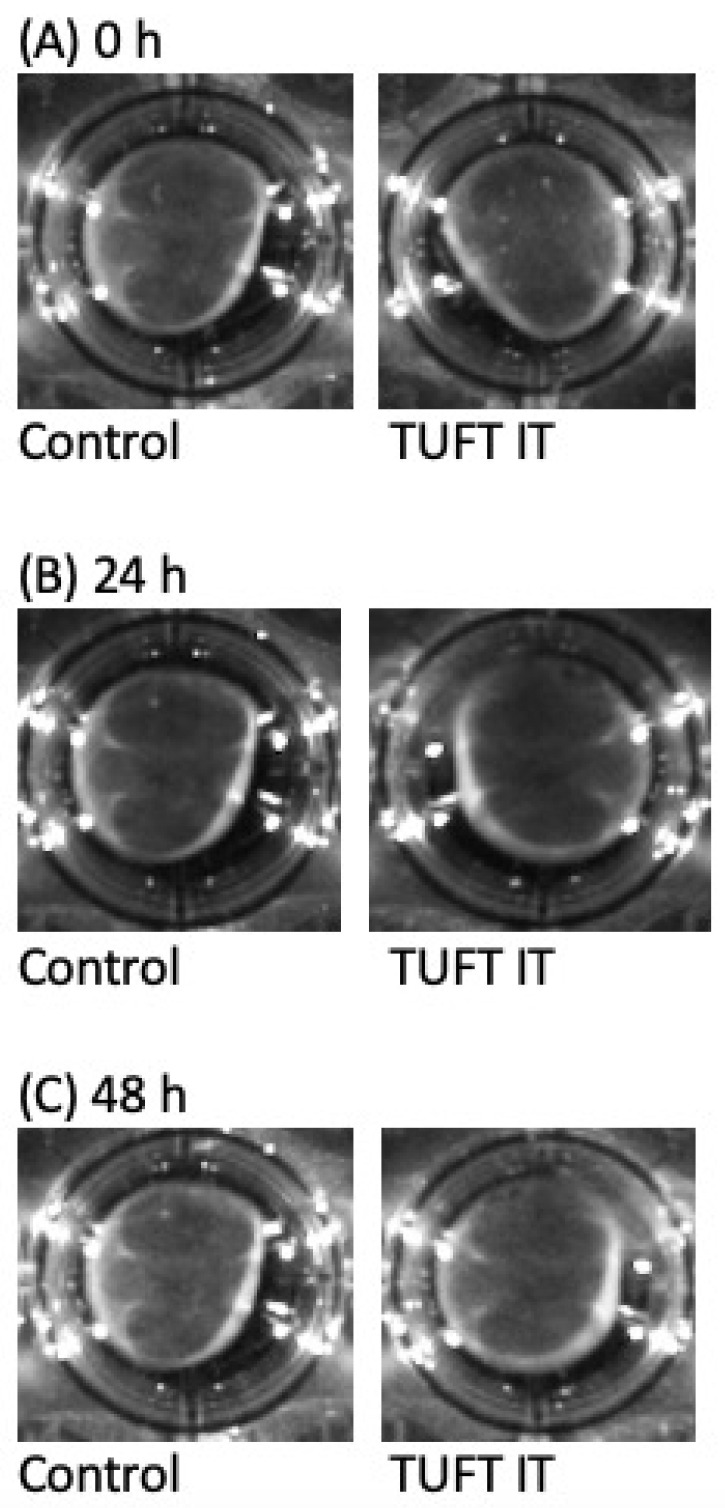
Pictures of the fibroblast without/with the gelatin-based hemostat during the contraction assay. No significant changes were visible at different time points 0 (**A**), 24 (**B**), and 48 h (**C**) after application. Control: group of fibroblasts where no hemostat was applied.

Comparing the contraction of the cell matrices, there was no significant difference between the control and the gelatin-based hemostat application group after 48 h. The contraction remained unchanged after gelatin-based hemostat application (Figure 2 and Figure 3).

The contraction of the cell matrices after photo processing (High 65,535, Low 51,765, Gamma 1.18) is shown for gelatin-based hemostat application at different time points (0, 24 and 48 h after application). There was no significant difference between the control (blue curve) and application group (green curve) within the 48 h time window. Cell matrix contraction remained unchanged after gelatin-based hemostat application (one-way ANOVA with Bonferroni correction, n = 12 per group). Control: Group of fibroblasts where no hemostat was applied.

### 2.3. Enzyme-Linked Immunosorbent Assay (ELISA)

**Figure 4:**
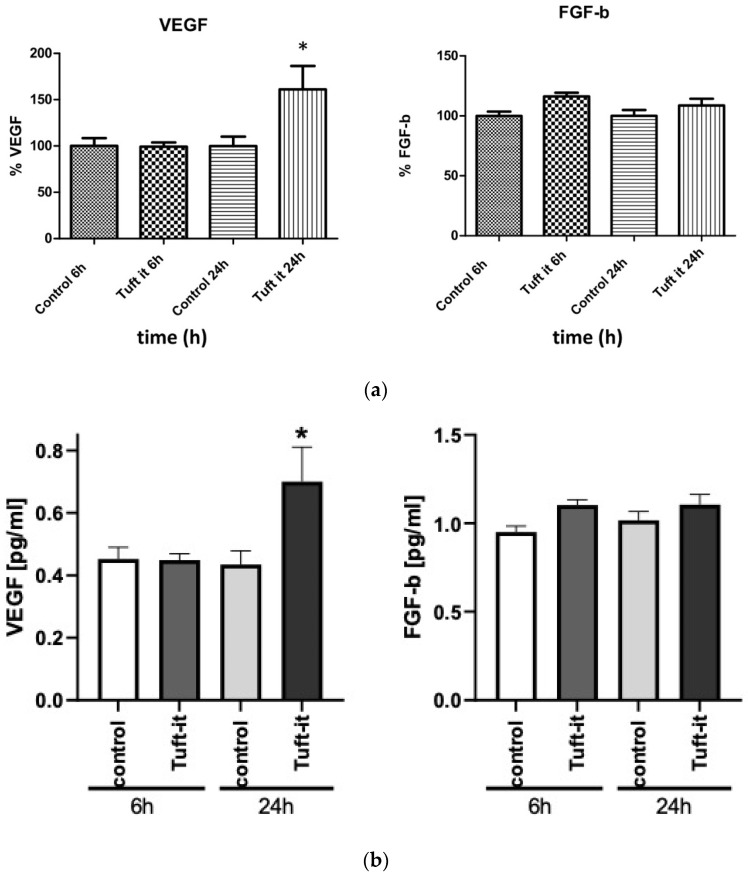
(**a**) Concentration of different growth factors (%) after gelatin-based hemostat application. This figure shows the concentrations of the different growth factors (VEGF and FGF-b) with and without gelatin-based hemostat application at different time points (after 6 and 24 h). VEGF showed a significant concentration increase under gelatin-based hemostat application after 24 h compared to the control (*p* < 0.05). Control: group of fibroblasts where no hemostat was applied. (**b**) The results of the expression of VEGF and FGF-b ELISA are presented in pg/mL (*p* < 0.05). Control: group of fibroblasts where no hemostat was applied. * (*p* < 0.05).

After applying gelatin-based hemostat for 6 h, there were no significant alterations observed in VEGF expression (0.452 pg/mL in the control group vs. 0.448 pg/mL in the hemostat group). After 24 h, VEGF production increased significantly by 61% compared to controls or to the 6 h application group (0.453 pg/mL in the control group vs. 0.697 pg/mL in the hemostat group, *p* < 0.05). The concentration of FGF-b did not show any significant changes after 24 h of gelatin-based hemostat application compared to controls (1017 pg/mL in the control group vs. 1.106 pg/mL in the hemostat group) (Figure 4a,b).

## 3. Discussion

### 3.1. Cell Proliferation

The application of gelatin-based hemostats resulted in a negative impact on cell proliferation at 7 and 14 days post-application. However, previous studies have reported inconsistent findings regarding the effect of gelatin-based products on cell proliferation. For example, gelatin application was found to stimulate embryonic mouse fibroblasts in vitro in a study by Orlova et al. (2014) [41] and a gelatin- and alginate-based hydrogel was shown to promote cell viability and proliferation in mouse fibroblasts after 7 and 14 days in a study by Zeng and Chen (2010) [42]. Similarly, an alginate hydrogel containing gelatin demonstrated immediate positive effects on cell viability and proliferation in normal human dermal fibroblasts compared to an alginate-only hydrogel in an in vitro study by Sarker et al. (2014) [43]. Additionally, a gelatin-containing wound dressing tested on porcine skin led to stronger fibroblast proliferation [44]. In contrast, our findings suggest that the gelatin-based hemostat tested here can inhibit fibroblast proliferation when left in the wound. It is important to note that in our experimental setting, the fibroblasts were left in the culture for 7 days without changing the cell culture medium, which could limit the availability of the necessary nutrients and space. Therefore, other factors such as stress, apoptosis, and cell arrest as a reaction to limited space and nutrients could also contribute to the observed inhibition of cell proliferation and may not necessarily be attributed to the gelatin-based hemostat application alone.

### 3.2. Cell Contraction

Furthermore, our results indicate that the gelatin-based hemostat did not have a significant effect on fibroblast contraction after 24 and 48 h of application when compared to controls. The effect of gelatin on wound contraction remains a topic of debate in the literature, with conflicting results reported. Some studies have demonstrated a positive effect on wound contraction. For example, an in vitro and in vivo study in Wistar rats showed faster skin wound contraction when treated with a combination of gelatin-nanofibers and polyvinyl alcohol hydrogel compared to controls without wound dressing [45]. Another in vitro study even showed an enhanced contraction of rat cardiac myocytes after gelatin-containing hydrogel application [46].

Additionally, an in vivo study on Göttingen mini pigs confirmed the positive effect of gelatin–collagen material on wound contraction [47] and a combined material of gelatin and chitosan demonstrated a positive effect on wound contraction after 14 days in another in vivo study in Wistar rats [48].

In contrast, an in vivo study in rats examining the effects of three intraperitoneal gelatin injections showed a delay in wound contraction after 7 and 14 days, likely due to a decrease in fibronectin concentration [49]. Regarding our results, the gelatin-based hemostat does not demonstrate a negative impact on fibroblast contraction.

### 3.3. Growth Factors

Based on the essential role of VEGF and FGF-b in wound healing described in the literature, we selected these cytokines for investigation in our study [3,50,51].

When compared to controls, VEGF expression was significantly increased by 61% after application of the gelatin-based hemostat for 24 h. FGF-b concentration increased by 16% after 24 h of gelatin-based hemostat application. Though the changes were not significant, they indicate that gelatin-based hemostat did not negatively influence FGF-b expression.

VEGF plays a crucial role in angiogenesis by promoting the proliferation of postcapillary endothelial cells through the production of NO and accumulation of cGMP, which is essential for the formation of well-perfused granulation tissue. This is supported by various studies, including those of Gale et al. and Morbidelli et al. [12,52]. A recent in vivo study with VEGF-overexpressing fibroblasts in mice resulted in a reduced wound area, stronger angiogenesis and the formation of granulation tissue, thereby emphasizing the potential of VEGF in wound healing [3]. This was further confirmed by an in vivo experiment in pig skin, showing promising results for wound healing with enhanced angiogenesis and collagen deposition when using a 3D-printed gelatin hydrogel patch combined with a VEGF-mimicking peptide [51].

FGF-b stimulates collagen synthesis, wound contraction, epithelialization as well as fibronectin and proteoglycan synthesis [14,15].

In an artificial dermis model, Kawai et al. investigated the effect of free FGF-b and FGF-b-impregnated gelatin microspheres on fibroblast proliferation and found that the latter significantly enhanced tissue regeneration when compared to the former [53].

Suzuki et al. (2013) utilized gelatin gel as a carrier material for growth factors such as TGF-ß and FGF-b, which led to improved angiogenesis and the development of granulation tissue [54].

Furthermore, Jinno et al. (2016) compared the use of FGF-b-treated collagen sponge and FGF-b-treated gelatin–collagen sponge in rats and found that the latter resulted in a significantly higher development of skin-like tissue [55]. In a recent study, the adipogenic effects of a collagen/gelatin sponge releasing FGF-b were investigated in the subcutis of mice, describing the material as a potential instrument in the treatment of soft tissue defects [56].

The mentioned investigations provide further evidence of gelatin’s compatibility as a carrier material for growth factors.

The effects on stromal fibroblasts cannot be fully investigated in our in vitro model, as it does not mimic in vivo circumstances comprising a variety of cell types involved in wound healing.

## 4. Conclusions

This study aimed to analyze the effect of a gelatin-based hemostat on central aspects of wound healing including fibroblast cell-proliferation, extracellular matrix contraction, b-FGF and VEGF levels as former studies showed contradicting results. Regarding our ELISA results as well as former studies, the gelatin-based material seems to be compatible with growth factors and even shows an increase in VEGF. Contraction was not impaired, while only fibroblast proliferation diminished at late time points when compared to controls. These results propose gelatin-based hemostats as a viable material for further surgical use. As an outlook, an experimental animal and human trial could reveal an extended range of clinical effects of gelatin-based hemostats on wound healing. Thus, further studies may clarify the clinical impact and usability of gelatin-based hemostats in an intra-operative setting.

## 5. Material and Methods

### 5.1. Hemostat GELITA TUFT-IT^®^

In this study, the gelatin-based hemostat (GELITA TUFT-IT^®^; GELITA MEDICAL GmbH, Eberbach, Germany) was investigated. According to the manufacturer’s protocol, the material is produced from 100% pure porcine gelatin, is pH-neutral, water-insoluble, and biodegradable, with complete absorbability achievable after 4 weeks.

To prepare the wells for further experiments, TUFT-IT (1 × 1 cm) was placed into each well with different application times of 5, 30 and 60 min, 24 h as well as 7 and 14 days.

The pieces of TUFT-IT were prepared by placing these on a sterile cloth and cutting them into pieces of 1 × 1 cm using sterile scissors and tweezers and stored in sterile Petri dishes. Afterwards, the well plates (with 12 wells per test group) were placed in a cell culture incubator until the times of measurement at 3, 6, 12, 24 h and 7 or 14 days, respectively.

### 5.2. Cell Culture

Human stromal fibroblasts (PromoCell GmbH, Heidelberg, Germany) were cultivated in Dulbecco’s Modified Eagle Media (DMEM) (Biochrom GmbH, Berlin, Germany) supplemented with 20% fetal bovine calf serum (Biochrom, Berlin, Germany) and 10 U/mL penicillin/streptomycin (PAN Biotech GmbH, Aidenbach, Germany). Fibroblasts were cultured at 37 °C and CO_2_ 5% (HERAcell240, Heraeus, Hanau, Germany) with regular media change. At 90% confluence, the cells were sub-cultured using 0.05% trypsin/0.02% ethylenediaminetetraacetic acid (EDTA) (PAN Biotech GmbH, Aidenbach, Germany). Passages 3 to 9 were used for experiments. Morphological cell assessment was performed using phase-contrast microscopy (Olympus CKX41, Olympus, Shinjuku, Japan).

### 5.3. Cell Proliferation

The cells were detached, centrifuged (5 min, 200× *g*, Thermo Fisher Scientific, Waltham, MA, USA), and resuspended in culture medium. A 30 µL aliquot of the cell suspension was mixed with 30 µL trypan blue in a 1.5 mL reaction container. Cell counting was performed using a Neubauer counting chamber and a light microscope, with the number of cells per milliliter being calculated using a specific formula.

### 5.4. Contraction Assay

The fibroblasts were seeded in a collagen I matrix (5 mg/mL rat tail collagen 1, ibidi GmbH, Planegg, Germany) consisting of 5 mg/mL rat tail collagen I. The mixture was pipetted into 24-well plates and supplemented with culture medium to a final volume of 300 μL. After gelation for 30 min in a cell culture incubator (HERAcell240, Heraeus, Hanau, Germany), 1 × 1 cm hemostat slices were added to the supernatant along with 2 mL of culture medium. Adherent matrices were detached after 24 h, photographed (ChemiDoc MP Imaging System, Bio Rad-Laboratories, Hercules, CA, USA), and placed back in the incubator for an additional 48 h with images taken at 24 and 48 h. The surface area of the matrices was calculated using Axio Vision software 40 × 64 V 4.9.1.0 (Carl Zeiss Microscopy GmbH, Jena, Germany).

### 5.5. Enzyme-Linked Immunosorbent Assay (ELISA)

The enzyme-linked immunosorbent assay (ELISA) was as far as possible conducted according to the protocol by R&D Systems for each DUOSet-ELISA-development system for the determination of VEGF and FGF basic.

Capture antibody and 100 µL of antibody solution were added to separate 96-well plates and incubated overnight. The plates were washed and blocked using reagent diluent (1% BSA in PBS, pH 7.2) for the VEGF- and FGF-basic-ELISAs. Meanwhile, the respective standard was diluted with reagent diluent (VEGF, FGF-basic) and a dilution series was produced according to manufacturer’s specifications.

The plates were then warmed, washed, and the detection antibody was diluted with reagent diluent according to the pre-defined concentrations (VEGF 100 ng/mL, FGF-basic 0.25 µg/mL) and 100 µL were pipetted into the respective wells. This was followed by a further incubation for 2 h.

After another round of washing, streptavidin-HRP was added and incubated for 30 min. The plates were washed again, substrate solution was added, and the reaction was stopped. Absorbance was measured at 450 nm using a plate reader VICTOR X4 by PerlkinElmer, Waltham, Massachusetts, USA, and a gradient of 0.9 was considered suitable after subtracting the absorption at 570 nm.

Each well of a 12-well cell culture plate was filled with 3 mL of a medium containing 1 × 10^5^ cells. The cell culture plates were then incubated overnight to provide adequate settling of the cells and adherence to the well floor. The following day medium was removed and replaced with fresh medium. Afterwards, the gelatin-based hemostat was added, and the expression levels of VEGF and FGF-b were evaluated at 6 and 24 h after application.

### 5.6. Statistical Analysis

Data are presented as mean ± SEM. Analysis was performed using GraphPad Prism 6.0 (San Diego, CA, USA). The one- and two-way ANOVA with Bonferroni test was used to test for significance. The significance level was set to *p* < 0.05.

## Abbreviations

FGF-b	Basic fibroblast growth factor
DMRM	Dulbecco’s phosphate-buffered saline
ECM	Extracellular matrix
EDTA	Ethylenediaminetetraacetic acid
ELISA	Enzyme-linked immunosorbent assay
NHDF	Normal human dermal fibroblast
PBS	Phosphate buffered saline
PDGF	Platelet-derived growth factor
TGFβ	Transforming growth factor β
TNFα	Tumor necrosis factor α
SEM	Standard error of the mean
VEGF	Vascular endothelial growth factor
